# Nb and Mn Co-Modified Na_0.5_Bi_4.5_Ti_4_O_15_ Bismuth-Layered Ceramics for High-Frequency Transducer Applications

**DOI:** 10.3390/mi13081246

**Published:** 2022-08-02

**Authors:** Dongming Fan, Huiyan Niu, Kun Liu, Xinhao Sun, Husheng Wang, Kefei Shi, Wen Mo, Zhishui Jian, Li Wen, Meng Shen, Tianlong Zhao, Chunlong Fei, Yong Chen

**Affiliations:** 1 Key Laboratory of Ferro & Piezoelectric Materials and Devices of Hubei Province, Hubei Collaborative Innovation Center for Advanced Organic Chemical Materials, Key Laboratory of Green Preparation and Application for Functional Materials, Ministry of Education, School of Physics and Electronic Science, Hubei University, Wuhan 430062, China; fandm99@163.com (D.F.); 13193650653@163.com (H.N.); kliu2020@163.com (K.L.); whs13057160921@163.com (H.W.); mhh20220330@163.com (W.M.); 2 School of Microelectronics, Xidian University, Xi’an 740071, China; xinhaosun@126.com (X.S.); fuka0902@stu.xidian.edu.cn (K.S.); clfei@xidian.edu.cn (C.F.); 3 Guangdong JC Technological Innovation Electronics Co., Ltd., Zhaoqing 526000, China; loong_jzs@163.com (Z.J.); wlapmz@163.com (L.W.)

**Keywords:** environmental friendliness, ultrasound transducers, ultrasound imaging, high-resolution imaging

## Abstract

Lead-free environmentally friendly piezoelectrical materials with enhanced piezoelectric properties are of great significance for high-resolution ultrasound imaging applications. In this paper, Na_0.5_Bi_4.5_Ti_3.86_Mn_0.06_Nb_0.08_O_15+y_ (NBT-Nb-Mn) bismuth-layer-structured ceramics were prepared by solid-phase synthesis. The crystallographic structure, micromorphology, and piezoelectrical and electromechanical properties of NBT-Nb-Mn ceramics were examined, showing their enhanced piezoelectricity (*d*_33_ = 33 pC/N) and relatively high electromechanical coupling coefficient (*k*_t_ = 0.4). The purpose of this article is to describe the development of single element ultrasonic transducers based on these piezoelectric ceramics. The as-prepared high-frequency tightly focused transducer (ƒ-number = 1.13) had an electromechanical coupling coefficient of 0.48. The center frequency was determined to be 37.4 MHz and the −6 dB bandwidth to be 47.2%. According to the B-mode imaging experiment of 25 μm tungsten wires, lateral resolution of the transducer was calculated as 56 μm. Additionally, the experimental results were highly correlated to the results simulated by COMSOL software. By scanning a coin, the imaging effect of the transducer was further evaluated, demonstrating the application advantages of the prepared transducer in the field of high-sensitivity ultrasound imaging.

## 1. Introduction

Ultrasound is becoming an increasingly important study area in acoustics due to its promising future. High-frequency ultrasound has a wide range of applications in biomedical imaging, non-destructive testing, and material micromechanical testing [1,2]. Recent years have witnessed a surge in scholarly interest in high-frequency ultrasound imaging technology with significant therapeutic potential. Ultrasound imaging has become one of the principal diagnostic techniques in modern clinical medicine because of its safety, intuition, flexibility, economy, and repeatability [3,4]. Compared to conventional medical ultrasound (2–15 MHz), high-frequency ultrasound sacrifices penetration depth for increased spatial resolution [5]. As a result, the significance of high-frequency ultrasound in clinical diagnostics and biomedical tissue research is indisputable [6].

As the primary component of high-frequency ultrasound technology, ultrasound transducers are essential for the overall performance of ultrasound systems. The performance of an ultrasonic transducer is limited by its piezoelectric material. Depending on the application scenario, it is significant to select the suitable piezoelectric material and design the piezoelectric layer. Piezoelectric ceramics are indispensable in the industrial production of ultrasonic transducers because of their low dielectric loss, high electromechanical conversion capability, and excellent mechanical properties [7]. Pb(Zr, Ti)O_3_ (PZT) is the preferred piezoelectric ceramic for the production of high-performance transducers because of its low manufacturing cost and high performance [8,9].

Nonetheless, the volatilization of lead oxide in PZT has resulted in tremendous environmental pollution during its manufacture and disposal. Therefore, environmentally friendly lead-free piezoelectric ceramics may be an attractive alternative. In recent years, researchers have demonstrated an increased interest in developing and applying lead-free piezoelectric ceramics [10,11,12,13]. Na_0.5_Bi_4.5_Ti_4_O_15_-based (NBT) piezoelectric ceramic materials have developed into a research focus because of their high Curie temperatures and good dielectric properties for harsh environment applications [14,15,16,17]. Nevertheless, pure NBT piezoelectric ceramics exhibit low piezoelectric and ferroelectric performance, which are incompatible with the requirements of general piezoelectric devices. It has previously been demonstrated that dopant-induced modifications can enhance the electrical properties of bismuth-layered ceramics. Numerous experimental studies have established that adding Mn, La, Co, Ba, Ce, or other rare earth elements significantly improve the performance of NBT ceramics [18,19,20,21,22,23,24]. In this paper, Nb_2_O_5_ and MnO_2_ are doped into NBT ceramics to promote the sintering process and improve their piezoelectric properties. In summary, improving material electrical properties is critical for developing ultrasonic transducers.

The past twenty years have seen increasing advances in highly functional sensors based on leadless ceramics. S.T.F. Lee et al. have developed 40 MHz lead-free transducers with insertion loss of −26 dB and a −6 dB bandwidth of 76.4% [25]. Can Wang et al. have prepared 1.615 MHz transducers with a −6 dB bandwidth of 56.25% based on Ca and Hf doped barium titanate ceramics [26]. Yi Quan et al. fabricated tightly focused KNN-based transducers, having a center frequency over 80 MHz and a −6 dB bandwidth of 52% [27]. Ultrasound imaging requires a higher resolution to improve the image quality [28,29,30,31]. It is widely believed that the center frequency and bandwidth of ultrasound transducers are critical parameters that affect the image quality [32]. Generally, increasing the center frequency results in optimizing spatial resolution while decreasing scanning depth [33]. Furthermore, the *ƒ*-number is a significant factor in influencing lateral resolution, which also needs to be considered in transducer design [34]. Numerous studies have begun to examine the performance of high-resolution ultrasound imaging in ophthalmology, dermatology, cardiology, and medical experiments involving small animals [35,36,37,38,39]. Fei et al. obtained a series of bio-microscopy images of zebrafish eyes using lithium niobate (LiNbO_3_) single-element ultrasonic transducers [40]. Zhang et al. have developed a transducer based on sodium bismuth titanate material to successfully capture images of skin on the back of a hand [41]. Zhang et al. reported a high-frequency miniature ultrasound transducer for intravascular imaging and performed imaging tests on porcine coronary arteries [42].

In this work, Na_0.5_Bi_4.5_Ti_3.86_Mn_0.06_Nb_0.08_O_15+y_ ceramics with high piezoelectric properties were fabricated by solid-phase synthesis. On this basis, tightly focused ultrasound transducers with a center frequency of 40 MHz were designed and manufactured. The electrical, acoustic, and imaging characteristics of the transducer were analyzed. The results of the relative experiment coincide well with the results simulated by PiezoCAD and COMSOL software. The study demonstrates that the transducers have potential applications in high-precision microscopic imaging. Research results have demonstrated that NBT-based lead-free piezoelectric ceramics are appealing materials for imaging applications involving high-frequency ultrasound.

## 2. Fabrication and Characterization of Piezoelectric Ceramics

### 2.1. Fabrication

NBT-Nb-Mn ceramics were obtained by using the solid-phase synthesis method. The raw materials used in this experiment were analytically pure Na_2_CO_3_ (99.9%), Bi_2_O_3_ (99.9%), TiO_2_ (99.9%), MnO_2_ (99.0%), and Nb_2_O_5_ (99.9%), which were weighed in accordance with their stoichiometric proportion compositions. The combined powders were ground for twelve hours in a ball grinder using zirconia balls and 60% ethanol. After ball milling and drying, the mixture was calcined at 800 °C for three hours to obtain the phase Na_0.5_Bi_4.5_Ti_4_O_15_. The ball-milling, drying, and grinding processes were repeated once to facilitate granulation. The powder was then pressed at 6–10 MPa with 9 wt% polyvinyl alcohol (PVA) binder. During the second sintering process, the thin cylinders were placed in a sealed crucible containing alumina and heated to 980 °C to minimize the volatilization of bismuth oxide, and then maintained for 3 h. The samples were approximately 11 mm in diameter and 1 mm in thickness. Both sides of the ceramic samples were painted with Ag electrodes. The NBT ceramics were polarized in silicone oil at 180 °C for thirty minutes under a direct current field of 9–10 kV/mm.

### 2.2. Characterization

The dielectric, electromechanical, and piezoelectric properties of the NBT-Nb-Mn ceramic samples are detailed in Table 1. Utilizing a quasi-static piezoelectric constant meter (ZJ-3D, Institute of Acoustics, Beijing, China), the piezoelectric coefficients *d*_33_ of the ceramic samples were measured. The dielectric constants were determined at room temperature using an LCR digital bridge meter (TH2830, Tonghui, Changzhou, China). The X-ray diffraction (XRD) pattern of the NBT ceramics was obtained by an X-ray diffractometer (Bruker D8 Advanced, Bruker, Germany). The crystalline structure of the ceramics was illustrated by a scanning electron microscope (SEM) (JEOL 6400, JEOL Ltd., Tokyo, Japan). The dielectric properties of the ceramics were measured at 1 MHz by an impedance analyzer (4192 A Hewlett Packard, HP Agilent, Santa Clara, CA). The impedance spectrum of the NBT ceramics was analyzed based on an impedance meter (WK6500B 1J65120B, Wayne Kerr Electronics, London, United Kingdom). The electromechanical coupling coefficient ($k_{t}$) can be calculated from the resonant and antiresonant frequency ($f_{r}$ and $f_{a}$) as follows:

(1) $$k_{t}=\sqrt{\frac{\pi }{2}\cdot \frac{f_{r}}{f_{a}}\tan\left(\frac{\pi }{2}\cdot \frac{f_{a}-f_{r}}{f_{a}}\right)}$$

**Figure 1:**
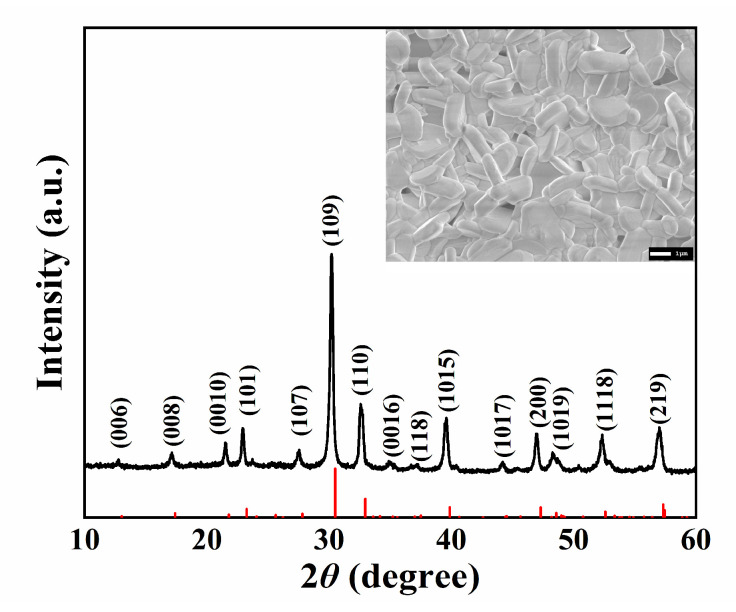
The XRD pattern and SEM image (embedded) of the NBT-Nb-Mn samples.

**Figure 2:**
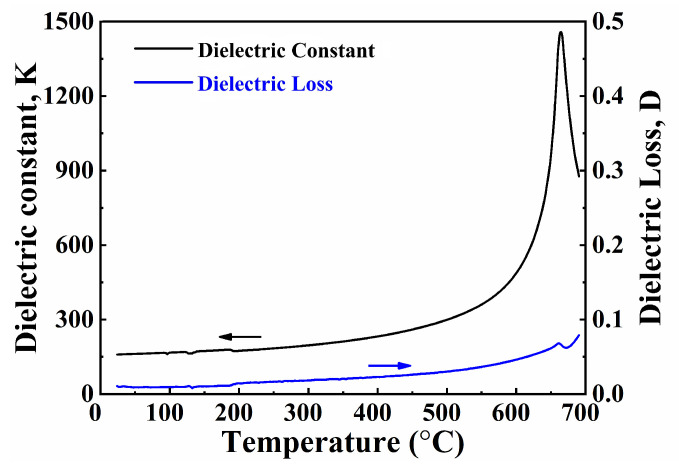
Dielectric constant K (black curve) and dielectric loss D (blue curve) of NBT-Nb-Mn ceramics sintered at 980 °C measured at 1 MHz.

As shown in Figure 1, the XRD pattern of the NBT-Nb-Mn powders matched the standard PDF card (PDF#74–1316) [43]. It can be seen that the doping of Nb and Mn did not change the phase structure of NBT ceramics. The SEM image exhibits flat plate-like grains from the surface of the ceramics, which is the typical morphology feature of bismuth-layered ceramics [21,22,23]. It appears from Figure 2 that the dielectric performance of the ceramics are reasonable under the test frequency of 1 MHz, and the Curie temperature reaches 660 °C and the dielectric loss tangent tan *δ* is 0.95% at room temperature, which meets the requirement of piezoelectric devices.

## 3. Design, Fabrication, and Characterization of Single-Element Ultrasound Transducers

### 3.1. Design and Simulation

**Figure 3:**
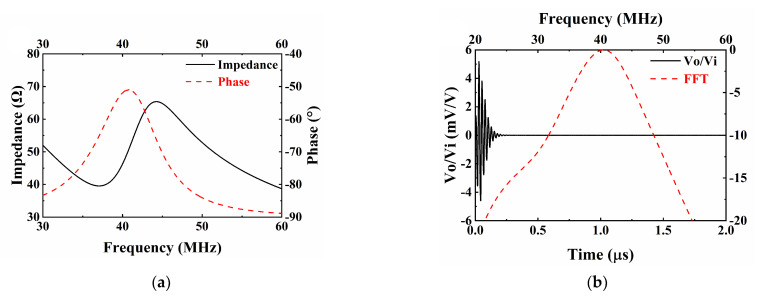
Modeling results of NBT-Nb-Mn single element transducer from KLM model-based simulation software PiezoCAD: (**a**) impedance simulation results; (**b**) pulse-echo simulation results.

On the basis of NBT-Nb-Mn piezoelectric ceramics, tightly focused transducers have been developed. The simulation software PiezoCAD (Sonic Concepts, Woodinville, WA, USA) based on the Krimholtz, Leedom, and Matthaei (KLM) model was used to design the ultrasound transducers. The critical material parameters used in the PiezoCAD software are summarized in Table 2. E-solder 3022 was selected as the backing, which has an acoustic impedance of 5.92 MRayls. Figure 3a,b illustrate the impedance and pulse-echo simulation results, respectively. The piezoelectric element has a size of 2 × 2 mm^2^ and a thickness of 48 µm. According to the simulation results, the designed transducer displays a center frequency of 40.1 MHz and a -6dB bandwidth of 20.1%.

**Figure 4:**
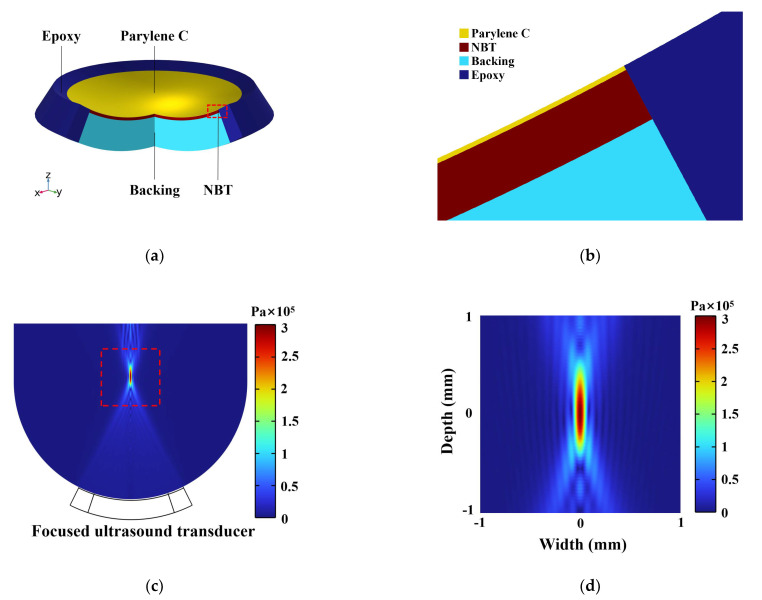

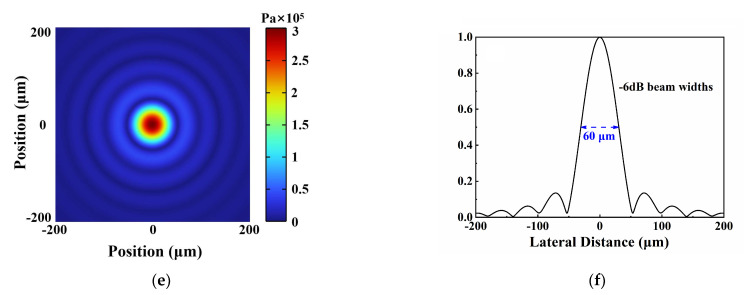
Modeling results of NBT-Nb-Mn single element transducer from FEM simulation software COMSOL: (**a**) schematic diagram of the three-dimensional structure of the transducer model; (**b**) cross-sectional schematic diagram of the three-dimensional structure of the transducer model; (**c**) cross-section of the acoustics field distribution in the water area; (**d**) magnitude map of the absolute acoustics pressure distribution in the *yz*-plane (*x* = 0); (**e**) magnitude map of the absolute acoustics pressure distribution in the *xy*-plane (*z* = 144 μm); (**f**) profile of normalized acoustics pressure amplitude along the lateral directions with -6 dB beam width indicated by blue line.

The finite element method (FEM) was used to calculate the distribution of absolute acoustics pressure produced by the transducer using COMSOL software. The piezoelectric ultrasonic transducer model was established in a two-dimensional axisymmetric coordinate system. The simulation parameters for the piezoelectric material were applied from the bismuth-layered material Bi_4_Ti_3_O_12_ [44]. The press-focused piezoelectric layer is approximated by a spherical shell with a radius of 3 mm, a solid angle of 0.74 sr, and a thickness of 48 μm. The constitutive relation of the piezoelectric layer is in stress–charge form, with 1 volt on the upper surface and grounded on the lower surface. The spherical shell was fixed in the center by epoxy, with a protective film on the upper surface and a backing layer on the lower surface. Epoxy and backing are both linear elastic materials with the boundary condition of a fixed constraint. The input parameters and model parameters for FEM simulation are listed in Table 3 and Table 4, respectively. The three-dimensional simulation model of the transducer and its cross-sectional schematic are shown in Figure 4a,b. The transducer was operated at 40 MHz and immersed in a hemispherical water area. The acoustic velocity in water was set at 1500 m/s and the default temperature of the model was 293.15 K. The physics used in this simulation include solid mechanics, the pressure acoustics frequency domain, and electrostatics, as well as multiphysics including the piezoelectric effect and the acoustic-structure boundary. The acoustic pressure distribution in the water area was calculated by FEM, and the lateral normalized acoustic pressure amplitude curve was drawn to calculate the theoretical lateral resolution of the transducer. Figure 4c illustrates the cross-section of the acoustics field distribution in the water area obtained by FEM. In the simulation, the focal length of the transducer was 3.14 mm. The sound pressure field of the yz-plane section (x = 0) and the xy-plane section (z = 144 μm) are depicted in Figure 4d,e, respectively. The normalized acoustic pressure amplitude curve along the lateral direction is shown in Figure 4f, and the −6dB lateral beamwidth reaches 60 μm.

### 3.2. Fabrication

**Figure 5:**
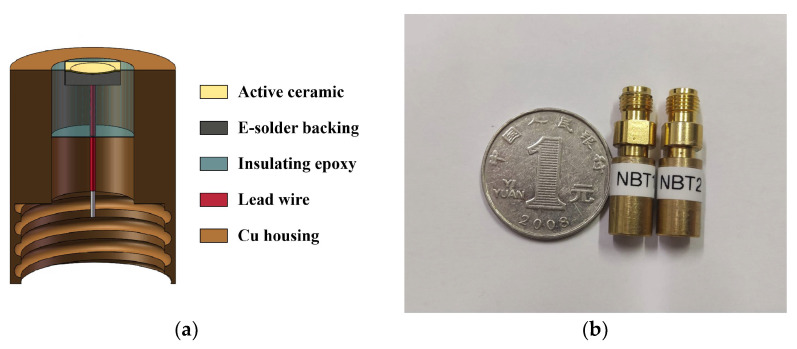
(**a**) Schematic diagram of the internal structure of the transducer; (**b**) end product image of the finished transducer.

Figure 5a shows the structural diagrams of the high-frequency transducers. Initially, paraffin waxes with a melting point of 60 °C were used as the adhesive between ceramics and flat glasses. The surface flatness error of flat glasses (5 × 5 cm^2^) was limited to 2 μm for controlling the thickness of ceramics precisely. The NBT ceramics were then manually ground to 48 μm using different meshes of sandpaper in order (400 meshes, 800 meshes, 1200 meshes, 2000 meshes). In this step, it was necessary to repeatedly measure the thickness of ceramic plates with a precision thickness gauge (ND 287, Heidenhain, Berlin, Germany) for preventing excessive grinding. Following this grinding process, 5 μm alumina powder and water were added for polishing. Using a magnetron sputtering system Desk V (Denton Vacuum, Moorestown, NJ, USA), both sides of the ceramics were plated with 300 nm thick Au electrodes. An E-solder 3022 (1.5 mm) layer was put on the backing side for absorbing ultrasound wave and vibration suppression in this study. With the backing layer dried and cured, the sample was cut into 2 × 2 mm^2^ square elements using a dicing saw (DAD 323, Disco, Tokyo, Japan). The backing layer was then attached to a copper wire. Afterwards, a brass housing was used to shell the piezoelectric element and was poured into the interior with epoxy. For creating an electrical path, the transducer’s front surface was sputtered with a Au electrode, and the transducer’s bottom was attached to SMA. For the purpose of improving the imaging performance, the transducer was press-focused in an oven at 65 °C using specially designed fixtures with high polished chrome/steel balls (diameter: 6 mm). Using a parylene deposition system (PDS 2010 Labcoator, Specialty Coating Systems, Indianapolis, IN, USA), parylene C was coated as a protective film on the transducer. The end product image of the ultrasound transducer is presented in Figure 5b.

### 3.3. Characterization

**Figure 6:**
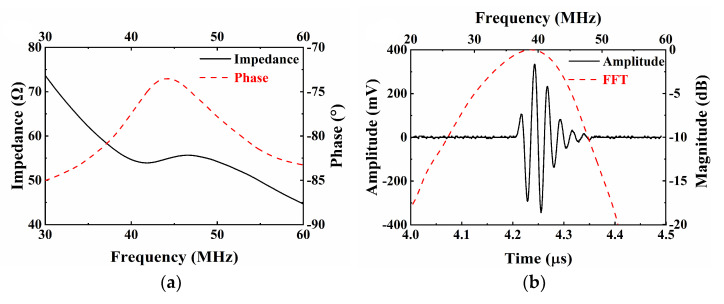
(**a**) Electrical impedance and phase of the NBT-Nb-Mn ultrasonic transducer; (**b**) pulse-echo response and normalized spectrum of the NBT-Nb-Mn ultrasonic transducers.

Figure 6a illustrates the impedance characteristics of the prepared ultrasonic transducer as a function of frequency. Impedance data were collected using an impedance analyzer (WK6500B, 1J65120B, Wayne Kerr Electronics, London, UK). Resonance frequency $f_{r}$ and antiresonance frequency $f_{a}$ were found at 41.9 and 46.7 MHz, respectively. The impedance at the center frequency was determined to be 54.9 Ω, which was consistent with the simulation results. The transmission efficiency of ultrasound can be improved through proper electrical matching, ensuring the excellent performance of ultrasonic transducers. The effective electromechanical coupling coefficient of the ultrasonic transducer was calculated to be 0.48 based on Equation (1).

The transducer was fixed on the adjustable clamp and immersed in deionized water. It was excited by a pulser-receiver (5073PR, Olympus, Allentown, PA, USA) to emit ultrasonic signals to a quartz plate. An oscilloscope (DSOX3024A, Keysight, Santa Rosa, CA, USA) displayed the received echo signal. Parameter settings include pulse repetition frequency of 1 kHz, the damping factor of 50 Ω, and a sampling rate of 4 GSa/s. As shown in Figure 6b, the pulse-echo test data were converted into a spectral profile by fast Fourier transform (FFT). The center frequency ($f_{c}$) and −6 dB bandwidth (BW) were calculated using the following formulas:

(2) $$f_{c}=\frac{f_{lower}+f_{upper}}{2}$$

(3) $$BW=\frac{f_{upper}-f_{lower}}{f_{c}}\cdot 100\%$$

where $f_{lower}$ and $f_{upper}$ are the corresponding frequency values at −6 dB. After experimental measurement and calculation, the measured centre frequency and −6 dB bandwidth were 37.4 MHz and 47.2%, respectively. The focal distance of the transducer was determined at 3.2 mm, and the ƒ-number was calculated as 1.13. The impedance and center frequency of the transducer are consistent with the results of the PiezoCAD simulation. The increase of −6 dB bandwidth can be attributed to spherical pressure focusing with an *ƒ*-number of 1.06 on the transducer. The unamplified peak-to-peak output voltage (V*_p-p_*) was 0.67 V*_p-p_*, which satisfies the imaging requirements.

Insertion loss is a crucial parameter to evaluate the performance of ultrasonic transducers. Insertion loss data of the transducer were collected by a signal generator (SMB 100A, Rohde & Schwarz, Munich, Germany) and a digital storage oscilloscope (DSOX3024A, Keysight, Santa Rosa, CA, USA). Insertion loss (*IL*) was calculated by the formula below:

(4) $$IL=20\log\frac{V_{echo}}{V_{emitted}}+1.9\;\mathrm{dB}+2.2\times 10^{-4}\times f^{2}\times 2d$$

Here, 1.9 dB and 2.2 × 10^−4^ (dB/cm∙MHz^2^) represent the loss of ultrasonic reflection by quartz and the attenuation coefficient of ultrasonic propagation in water, respectively. f is the excitation frequency in the insertion loss test and d is the focal length of the transducer. On the basis of measurement results, the $V_{echo}$ of the ultrasonic transducer at 37.4 MHz was 27.1 mV and the $V_{emitted}$ was 2.81 V. The insertion loss was calculated as −36.3 dB.

**Figure 7:**
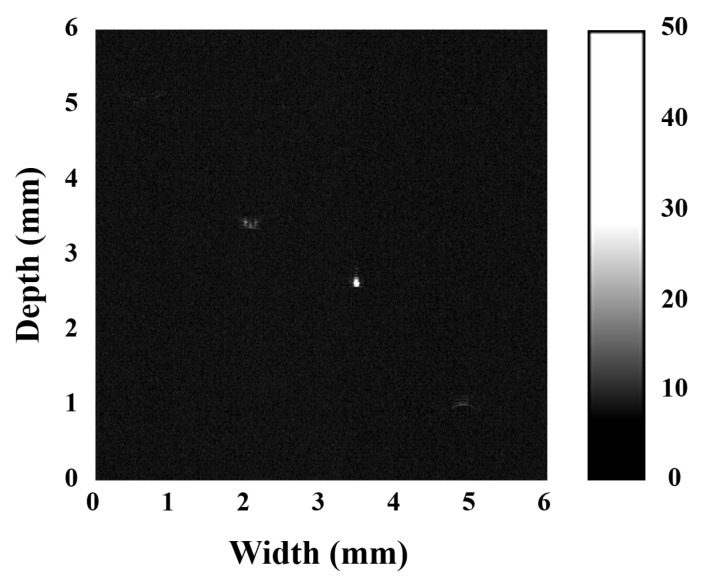
Wire phantom UBM images obtained by the 37 MHz NBT-Nb-Mn single element transducer.

**Figure 8:**
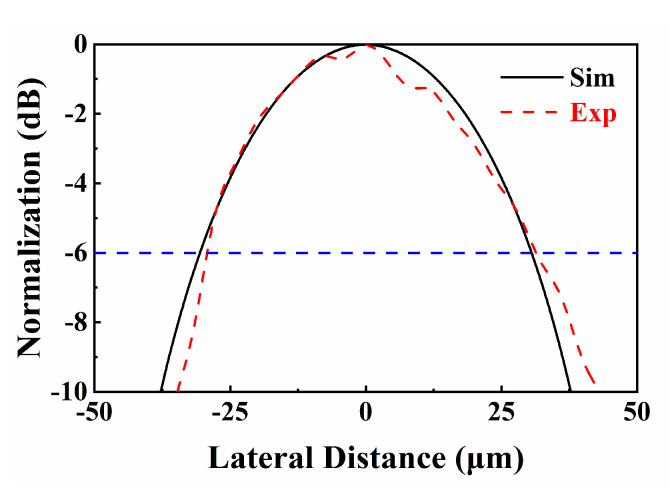
Comparison of the amplitude of simulation and experimental data for lateral line spread function.

Finally, the transducer was subjected to an imaging test. There are two essential types of spatial resolution in ultrasound imaging for focused ultrasound transducers: axial resolution ($R_{axial}$) and lateral resolution ($R_{lateral}$), which can be described in terms of the following formulas:

(5) $$R_{axial}=\frac{\lambda }{2BW}$$

(6) $$R_{lateral}=\lambda \cdot \left(\frac{L}{D}\right)=\lambda \times f_{\mathrm{number}}$$

where *λ* is the wavelength of the ultrasonic wave, *BW* and *L* are the bandwidth and focal length of the transducer, *D* is the diameter/diagonal length of the piezoelectric element, and the ratio of *L* to *D* is defined as the *ƒ*-number. Theoretical lateral resolution of the transducer was 43 μm. On the basis of the UBM (ultrasound bio-microscopy) system, the actual spatial resolution of the transducer was assessed by B-mode imaging 25 μm tungsten wires. Four tungsten wires were wound on a wire phantom, spaced about 1.5 mm apart axially and transversally. The focus of the transducer was aligned to the tungsten wire near the center with a distance of 3.2 mm. The UBM system consists of three components: a pulser-receiver, a motor controller, and an automated fixture. The pulser-receiver (DPR-500, JSR ultrasonics, New York, NY, USA) was used to generate electrical impulses that are applied to a transducer causing the transducer to emit an ultrasound pulse. The transducer was fixed on the fixture, and the motor drove the transducer to perform mechanical linear scanning of the wires through program control at 4 μm steps. As shown in Figure 7, the raw RF data from the reflected echo signals were then processed by MATLAB to produce an ultrasound image. It can be found that the image quality was reasonable and three of the wires were clearly visible. As the transducer was tightly focused, the acoustic energy at the focus reached its maximum, which makes the middle wire at focus the clearest. Figure 8 provides a comparison plot between the simulated (from Figure 4f) and experimental data of the lateral line spread function. Experiment results show that the transducer exhibited −6 dB lateral resolution of 56 μm. The measured results show that the experimental and simulation results are in good agreement. High-frequency ultrasound transducers offer significant advantages in resolution over conventional clinical ultrasound.

### 3.4. Ultrasound Microscope Imaging

**Figure 9:**
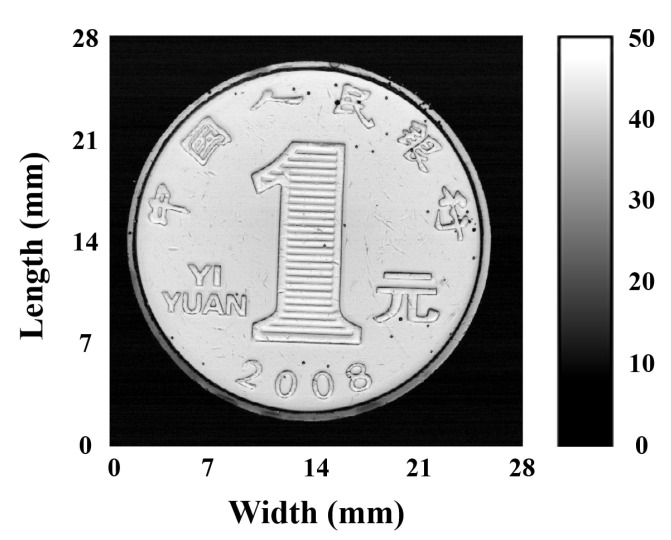
C-mode scanning images of the RMB coin.

An RMB one yuan coin was scanned to evaluate the imaging effect of the transducer. The imaging experimental process was performed on the UBM system. The coin was immersed in deionized water and kept parallel to the transducer. C-mode imaging tests were conducted with a scanning width and length of 28 mm, corresponding scan steps of 4 and 50 μm, and a minimum sampling rate of 160 MHz. As illustrated in Figure 9, the imaging results presented a clear image of the RMB coin.

Air bubbles caused a few black spots in the water. The results of this study have important implications for imaging small biological tissues with a shallow penetration depth requirement.

## 4. Conclusions

Lead-free piezoelectric materials are very critical for the development of environmentally friendly ultrasound transducers. In this work, lead-free bismuth-layered structural ceramics were modified by Nb and Mn doping. On this basis, focused ultrasound transducers for high-frequency imaging were designed and prepared.

The ceramic Na_0.5_Bi_4.5_Ti_3.86_Mn_0.06_Nb_0.08_O_15+y_ was synthesized using conventional solid-phase synthesis. XRD results showed that the doping of Nb and Mn did not change the phase structure of NBT ceramics. SEM images also show that the sample grains conform to the typical characteristics of bismuth-layered ceramics. Dielectric, piezoelectric, and electromechanical property test results exhibit the enhancement of material performance. The *d*_33_ of the ceramic sample reached 33 pC/N, and the *k*_t_ was calculated to be 0.4.

High-frequency ultrasonic transducers were designed and developed on the basis of this material. Impedance spectra, pulse-echo response, as well as absolute acoustic pressure distributions of the transducers were simulated using PiezoCAD and COMSOL software, respectively. The as-prepared, tightly focused transducer has a lateral resolution of 56 μm, with a center frequency of 37.4 MHz and a −6 dB bandwidth of 47.2%. It is consistent with the simulation results. Additionally, ultrasound imaging of RMB coins demonstrated the excellent imaging effect of the transducer. The experimental results provide an alternative to lead-free piezoelectric ultrasonic transducers and reveal the application potential of NBT materials in high-frequency ultrasonic imaging.

## Figures and Tables

**Table 1:** Dielectric, electromechanical, and piezoelectric properties of piezoelectric materials.

Property	NBT-Nb-Mn
Curie temperature T_c_	660 °C
Relative permittivity *ε*_33_^T^	159
Dielectric loss tangent tan δ	0.95%
Thickness electromechanical coupling *k*_t_	0.4
Piezoelectric coefficient *d*_33_	33 pC/N

**Table 2:** Parameters of piezoelectric materials used for PiezoCAD simulation modeling.

Property	NBT-Nb-Mn
Longitudinal velocity *υ*	3910 m/s
Density *ρ*	6430 kg/m^3^
Acoustic impedance *Z*	25.1 MRayl
Clamped relative dielectric constant *ε*_r_	97.37
Dielectric loss tangent tan *δ*	0.95%
Thickness electromechanical coupling *k*_t_	0.4
Piezoelectric coefficient *d*_33_	33 pC/N

**Table 3:** Parameters of materials used for finite element analysis.

Property	Unit	Esolder-3022	Epoxy	Parylene C
Pressure-wave speed *c_p_*	m/s	1850	2650	2200
Acoustic impedance *Z*	MRayl	5.92	3.05	2.6
Density *ρ*	kg/m^3^	3200	1150	1180

**Table 4:** The finite element model parameters of the transducer.

Parameters	Description
Piezoelectric layer shape	Spherical shell
Piezoelectric layer thickness	48 μm
Backing thickness	500 μm
Epoxy thickness	552 μm
Parylene C thickness	4 μm

## Data Availability

The data presented in this study are available on request from the corresponding author.
